# Three Cases of Primary Hepatic Neuroendocrine Tumor Treated with Surgical Resection: A Case Series and Literature Review

**DOI:** 10.70352/scrj.cr.26-0333

**Published:** 2026-10-16

**Authors:** Takanari Kobayashi, Sunao Fujiyoshi, Shunsuke Shichi, Yuzuru Sakamoto, Takeshi Aiyama, Akihisa Nagatsu, Norio Kawamura, Masaaki Watanabe, Ryoichi Goto, Tatsuhiko Kakisaka, Kenjiro Kato, Takuya Otsuka, Akinobu Taketomi

**Affiliations:** 1 Department of Gastroenterological Surgery 1, Graduate School of Medicine, Hokkaido University, Sapporo, Hokkaido, Japan; 2 Department of Surgical Pathology, Hokkaido University Hospital, Sapporo, Hokkaido, Japan

**Keywords:** primary hepatic neuroendocrine tumor, follow-up protocol, treatment strategy

## Abstract

**INTRODUCTION:**

Primary hepatic neuroendocrine tumors (PHNETs) are extremely rare, accounting for approximately 0.3% of all neuroendocrine tumors (NETs). Because the liver is the most common metastatic site of NETs, distinguishing PHNET from metastatic disease is often challenging. Optimal treatment strategies and follow-up protocols for PHNET have not been well established. We report 3 cases of PHNET treated with surgical resection.

**CASE PRESENTATION:**

Case 1 involved a 42-year-old woman with a liver tumor detected during a routine medical examination. Laparoscopic partial hepatectomy was performed under the preoperative diagnosis of hepatic adenoma or hemangioma. Histopathological examination revealed a NET, grade 2. The patient developed intrahepatic recurrence 5 years and 8 months after the initial surgery and underwent repeat hepatectomy. Case 2 involved a 72-year-old woman diagnosed with PHNET (grade 2) by liver biopsy. Laparoscopic extended posterior sectionectomy was performed. Intrahepatic recurrence occurred 3 years and 9 months later, and repeat hepatectomy was performed. Case 3 involved a 50-year-old man diagnosed with NET (grade 1) by endoscopic US-guided fine-needle aspiration. Extended left hepatectomy with partial resection of segment 8 was performed. He remains recurrence-free. In all cases, comprehensive examinations, including endoscopy, somatostatin receptor imaging (SRI), and PET, revealed no evidence of extrahepatic primary lesions, leading to the diagnosis of PHNET.

**CONCLUSIONS:**

PHNET is a rare tumor that may recur several years after curative resection. Careful long-term follow-up is therefore required, and repeat surgical resection may be an effective treatment option for selected patients with recurrent disease.

## Abbreviations

CD56
: cluster of differentiation 56
CDX-2
: caudal type homeobox 2
ENETS
: European Neuroendocrine Tumor Society
ESMO
: European Society for Medical Oncology
EUS-FNA
: endoscopic ultrasound-guided fine-needle aspiration
FDG
: fluorodeoxyglucose
Gd-EOB-MRI
: gadoxetic acid-enhanced MRI
HBcAb
: hepatitis B core antibody
HBsAb
: hepatitis B surface antibody
HBsAg
: hepatitis B virus surface antigen
ICGR15
: indocyanine green retention rate at 15 min
INSM1
: insulinoma-associated protein 1
NEC
: neuroendocrine carcinoma
NEN
: neuroendocrine neoplasm
NET
: neuroendocrine tumor
PHNET
: primary hepatic neuroendocrine tumor
PIVKA-II
: protein induced by the absence of vitamin K
PRRT
: peptide receptor radionuclide therapy
SPECT
: single photon emission CT
SRI
: somatostatin receptor imaging
SSTR
: somatostatin receptor
SUVMax
: standardized uptake value maximum
TTF-1
: thyroid transcription factor-1
WHO
: World Health Organization

## INTRODUCTION

NETs most commonly originate from the pancreas, gastrointestinal tract, or lungs. In contrast, PHNETs are extremely rare, accounting for approximately 0.3% of all NETs.^1)^ The optimal treatment strategy and follow-up protocols have not yet been established. Herein, we report 3 cases of PHNET treated with surgical resection, along with a review of the relevant literature.

## CASE PRESENTATION

### Case 1

A 42-year-old woman was referred to our hospital for evaluation of a liver tumor that had been detected during a routine abdominal ultrasonographic examination in X-2. She had no symptoms. Her past medical history and family history were unremarkable. At that time, the lesion was suspected to be a hepatocellular adenoma or hemangioma, and the patient was followed up conservatively. In X, because the tumor showed a tendency to increase in size, surgical resection was planned. On admission, her height was 168 cm, and body weight was 45 kg. Physical examination revealed a soft and non-distended abdomen without tenderness. Laboratory findings on admission were as follows: total bilirubin 0.9 mg/dL, albumin 4.7 g/dL, prothrombin time 10.9 s, and activated partial thromboplastin time 28.7 s. Viral hepatitis markers showed negative HBsAg, positive HBsAb, and negative HBcAb. Tumor markers were within normal limits (alpha-fetoprotein 6.3 ng/mL and PIVKA-II 35 mAU/mL). The ICGR15 was 8.0%. Liver function was classified as liver damage grade A and Child–Pugh class A (5 points). Contrast-enhanced CT revealed a 1.8 cm nodule in segment 2 of the liver with slight enhancement from the portal venous phase to the equilibrium phase. In addition, multiple hepatic hemangiomas were observed in segments 3, 6, and 8 (**Fig. 1A**). On Gd-EOB-MRI, the lesion in segment 2 showed no remarkable early enhancement and appeared hypointense compared with the surrounding liver parenchyma in the delayed phase. Decreased uptake was observed in the hepatobiliary phase (**Fig. 1B**). Upper and lower gastrointestinal endoscopy revealed no tumor lesions in the esophagus, stomach, duodenum, or colon. Based on a preoperative diagnosis of hepatocellular adenoma or hepatic hemangioma, laparoscopic partial resection of segment 2 was performed. Histopathological examination revealed proliferation of small atypical cells with round nuclei showing coarse chromatin and weakly eosinophilic cytoplasm in trabecular, glandular, and ribbon-like patterns. The mitotic count was 1 per 10 high-power fields, and the Ki-67 labeling index was 3.6%. Immunohistochemically, the tumor cells were positive for CD56, synaptophysin, and chromogranin A (**Fig. 2**). According to the 2019 WHO classification, the tumor was diagnosed as a NET, grade 2. The surgical margin was negative. To investigate the possible primary origin, additional immunohistochemical staining for CDX-2, TTF-1, islet-1, serotonin, somatostatin, gastrin, insulin, and glucagon was performed; however, no significant positive findings were observed. After the pathological diagnosis was established, ^111^In-pentetreotide scintigraphy and FDG-PET were performed, but no abnormal uptake suggestive of an extrahepatic primary lesion was identified. Therefore, the tumor was considered to be a PHNET. At 5 years and 8 months after the initial surgery, recurrence was detected in segment 4 of the liver, and robot-assisted partial resection of segment 4 was performed. Histopathological examination of the recurrent lesion showed a mitotic count of 2 per 10 high-power fields and a Ki-67 labeling index of 9.4%, consistent with NET, grade 2, and compatible with metastatic recurrence. At 2 months after the second surgery, the patient remains alive without treatment and with no evidence of recurrence.

**Fig. 1:**
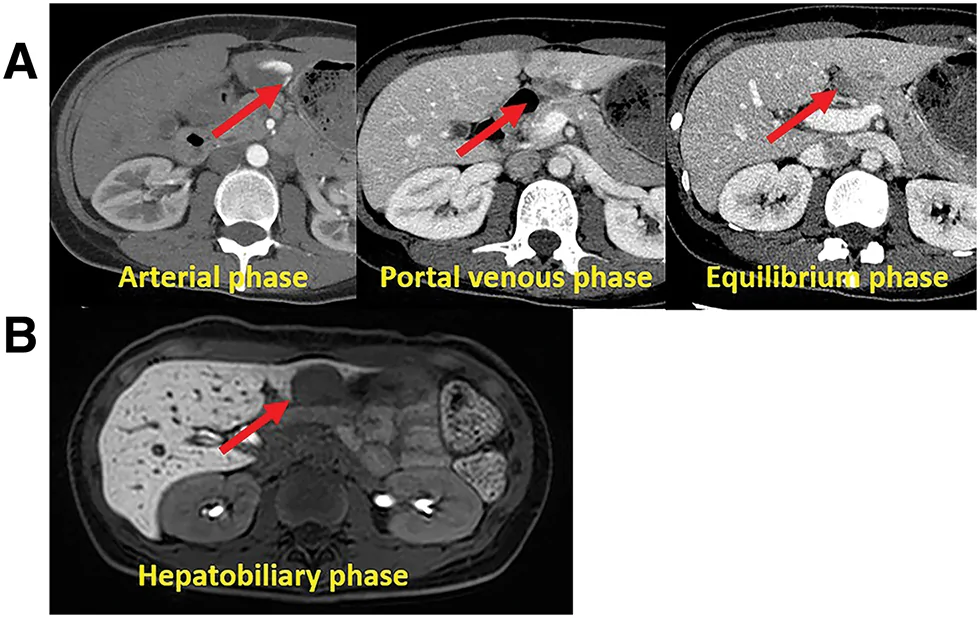
Contrast-enhanced CT and MRI of Case 1. Contrast-enhanced CT revealed an 18-mm nodule in segment 2 of the liver with slight enhancement from the portal venous phase to the equilibrium phase (**A**). On Gd-EOB-MRI, decreased uptake was observed in the corresponding lesion in the hepatobiliary phase (**B**). All arrows indicate tumors. Gd-EOB-MRI, gadoxetic acid-enhanced MRI

**Fig. 2:**
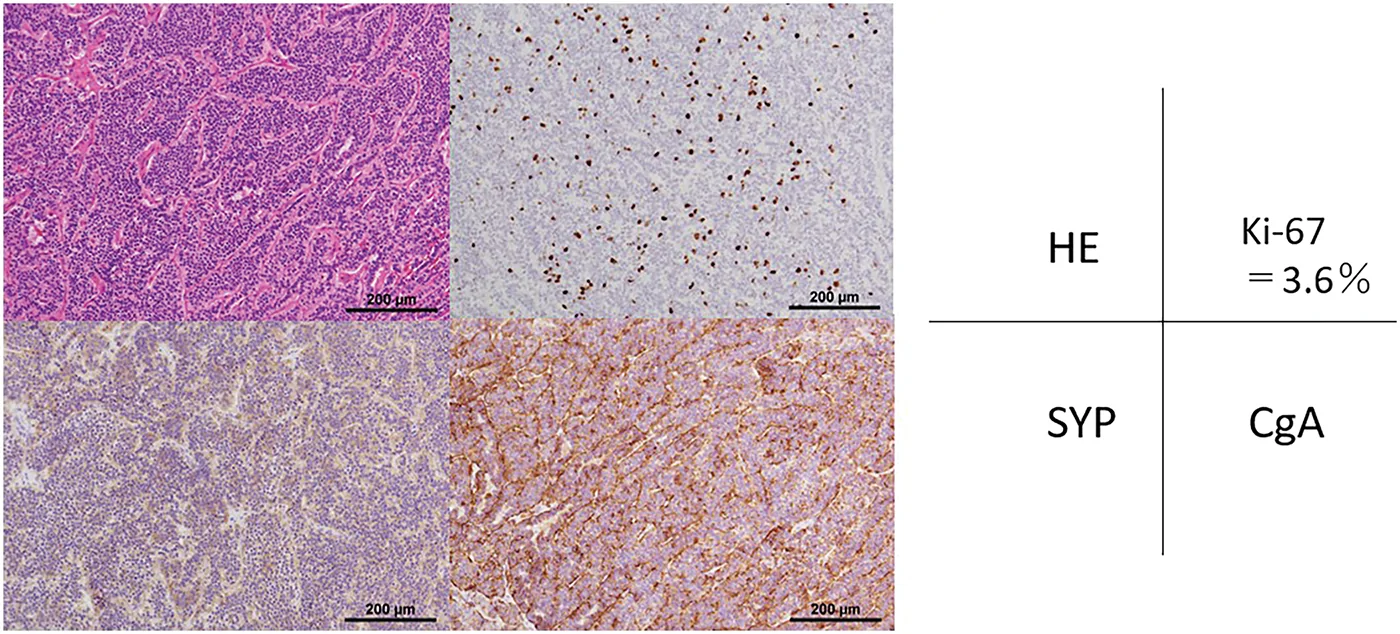
Pathological images of Case 1. Small atypical cells with round nuclei showing coarse chromatin and weakly eosinophilic cytoplasm proliferated in trabecular, glandular, and ribbon-like patterns. The Ki-67 labeling index was 3.6%, and immunohistochemistry showed positivity for CD56, SYP, and CgA. CD56, cluster of differentiation 56; CgA, chromogranin A; HE, hematoxylin-eosin; SYP, synaptophysin

### Case 2

A 72-year-old woman was referred to our department for surgical treatment of PHNET. She had no symptoms. Her past medical history included appendicitis treated by appendectomy at 20 years of age, left upper lobe lung cancer resected at 71 years of age, hypertension, and dyslipidemia. Her family history included pancreatic cancer in her mother and gastric cancer in her sister. In X-2, a liver tumor was detected on abdominal ultrasonography. Detailed examination revealed tumors in segments 6 and 8 of the liver. A biopsy was performed, and the lesion was diagnosed as PHNET (grade 2). She was referred to our department in X, and surgical resection was planned. On admission, her height was 154 cm, and body weight was 70 kg. Physical examination revealed a flat, soft abdomen without tenderness. A surgical scar was noted in the right lower abdomen. Laboratory findings on admission were as follows: total bilirubin 0.5 mg/dL, albumin 4.0 g/dL, prothrombin time 11.2 s, and activated partial thromboplastin time 31.2 s. All viral hepatitis markers were negative. Tumor markers were within normal limits (alpha-fetoprotein 3.0 ng/mL and PIVKA-II 16 mAU/mL). The ICGR15 was 16.5%. Liver function was classified as liver damage grade A and Child–Pugh class A (5 points). Contrast-enhanced CT revealed 3 early-enhancing nodules measuring 1.8, 1.5, and 0.8 cm: 1 in segment 6 and 2 in segment 8 of the liver (**Fig. 3A**). On Gd-EOB-MRI, all lesions identified on CT showed decreased uptake in the hepatobiliary phase (**Fig. 3B**). Upper and lower gastrointestinal endoscopy revealed a submucosal tumor on the posterior wall of the gastric antrum. No tumor lesions were identified in the esophagus, duodenum, or colon. ^111^In-pentetreotide scintigraphy demonstrated abnormal uptake in 1 lesion in segment 6 and 1 lesion in segment 8 of the liver (**Fig. 3C**). No abnormal uptake was observed in any other organs. FDG-PET showed no abnormal uptake. Based on the preoperative diagnosis of PHNET (grade 2), laparoscopic extended posterior sectionectomy was performed. Because all 3 lesions were located predominantly within the posterior sector, with some extending into segment 8, securing adequate surgical margins by multiple partial hepatectomies was considered technically difficult. Therefore, laparoscopic extended posterior sectionectomy with concomitant resection of the right hepatic vein was performed to achieve complete removal of all tumors with sufficient surgical margins. Histopathological examination revealed atypical cells with enlarged nuclei showing coarse chromatin and amphophilic cytoplasm, forming solid nests of various sizes. The mitotic count was 2 per 10 high-power fields, and the Ki-67 labeling index was 1.04%. Immunohistochemically, the tumor cells were positive for CD56, synaptophysin, and chromogranin A (**Fig. 4**). According to the 2019 WHO classification, the tumor was classified as NET G2 based on the mitotic count of 2 per 10 high-power fields, despite a Ki-67 labeling index of 1.04%. The surgical margin was negative. At 3 years and 9 months after the initial surgery, 2 recurrent lesions were detected in the left lateral section of the liver. No increase in the size of the gastric submucosal tumor was observed. Laparoscopic partial resection of segments 3 and 4 was performed. Histopathological examination revealed almost no identifiable mitotic figures and a Ki-67 labeling index of 1%, leading to a diagnosis of PHNET (grade 1), which was compatible with metastatic recurrence. At 7 months after the second surgery, the patient remains alive without treatment and with no evidence of recurrence.

**Fig. 3:**
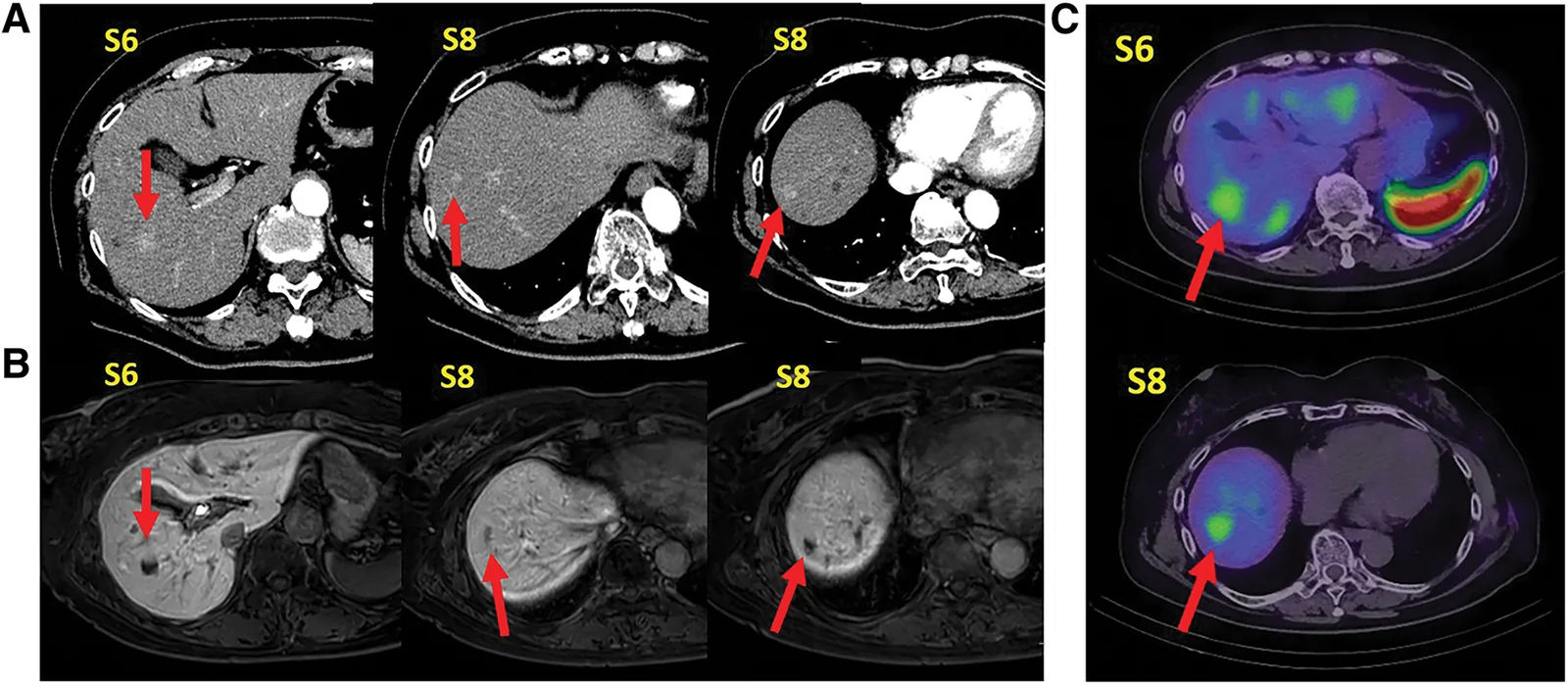
Contrast-enhanced CT and MRI of Case 2. Contrast-enhanced CT revealed 3 early-enhancing nodules measuring 18, 15, and 8 mm: 1 in segment 6 and 2 in segment 8 of the liver (**A**). On Gd-EOB-MRI, these lesions showed decreased uptake in the hepatobiliary phase (**B**). ^111^In-pentetreotide scintigraphy demonstrated abnormal uptake in 1 lesion in segment 6 and 1 lesion in segment 8 (**C**). All arrows indicate tumors. Gd-EOB-MRI, gadoxetic acid-enhanced MRI

**Fig. 4:**
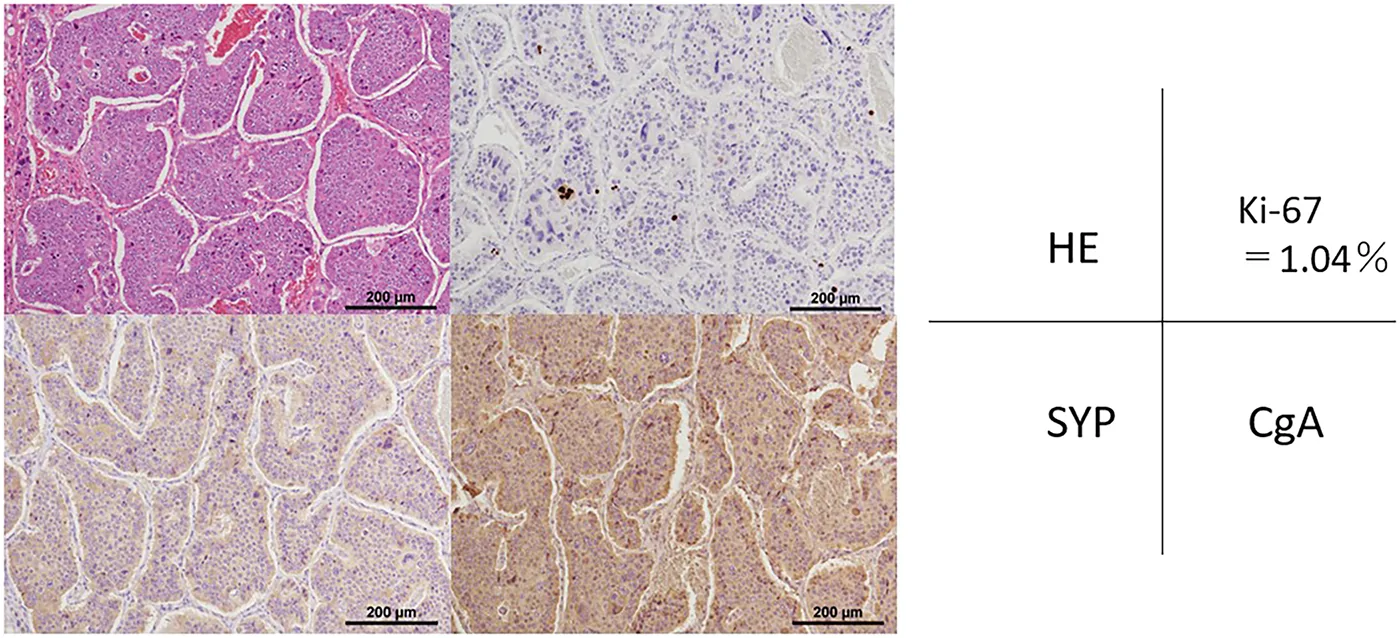
Pathological images of Case 2. Atypical cells with enlarged nuclei showing coarse chromatin and amphophilic cytoplasm formed solid nests of various sizes. The Ki-67 labeling index was 1.04%, and immunohistochemistry showed positivity for CD56, SYP, and CgA. CD56, cluster of differentiation 56; CgA, chromogranin A; HE, hematoxylin-eosin; SYP, synaptophysin

### Case 3

A 50-year-old man was referred to our department for surgical treatment of a primary hepatic neuroendocrine tumor. He had no symptoms. His past medical history included gastric cancer, pathological stage IIA, for which he had undergone total gastrectomy and cholecystectomy at 40 years of age, as well as hypertension. A liver tumor was detected on abdominal ultrasonography during a routine health checkup. Detailed examination revealed a hypervascular tumor in segment 1 of the liver. EUS-FNA was performed, and the lesion was diagnosed as a NET, grade 1. He was referred to our department in the same year, and surgical resection was planned. On admission, his height was 169 cm, and body weight was 90 kg. Physical examination revealed a flat, soft abdomen with an upper midline and transverse surgical scars. Laboratory findings on admission were as follows: total bilirubin 0.4 mg/dL, albumin 4.4 g/dL, prothrombin time 10.6 s, and activated partial thromboplastin time 23.8 s. All viral hepatitis markers were negative. Tumor markers were within normal limits (alpha-fetoprotein 2.0 ng/mL and PIVKA-II 39 mAU/mL). The ICGR15 was 9.4%. Liver function was classified as liver damage grade A and Child–Pugh class A (5 points). Contrast-enhanced CT revealed 5 early-enhancing nodules: 1 in segment 1, 2 in segment 3, and 2 in segment 8 of the liver, measuring 5, 2, 2, 1, and 1 cm, respectively (**Fig. 5A**). On Gd-EOB-MRI, all lesions identified on CT showed decreased uptake in the hepatobiliary phase (**Fig. 5B**). Upper and lower gastrointestinal endoscopy revealed no tumor lesions in the esophagus, stomach, duodenum, or colon. ^111^In-pentetreotide scintigraphy demonstrated abnormal uptake in 1 lesion in segment 1 and 1 lesion in segment 3 of the liver (**Fig. 5C**). FDG-PET demonstrated increased uptake in segment 1 (SUVmax, 7.9) and segment 3 (SUVmax, 6.2) (**Fig. 5D**). Based on the preoperative diagnosis of PHNET (grade 1), extended left hepatectomy with partial resection of segment 8 was performed. No peritoneal dissemination was observed, and intraoperative ultrasonography identified no lesions other than those detected preoperatively. Histopathological examination revealed proliferation of atypical cells forming rosette-like and glandular structures. The mitotic count was 4 per 10 high-power fields, and the Ki-67 labeling index was 10%. Immunohistochemically, the tumor cells were positive for INSM1 (**Fig. 6**). According to the 2019 WHO classification, the tumor was diagnosed as a NET, grade 2. Regarding the surgical margin, tumor components with thermal degeneration were observed, and involvement of the dissection margin could not be ruled out. The patient has been followed for 6 months after surgery without any treatment, and no recurrence has been observed.

**Fig. 5:**
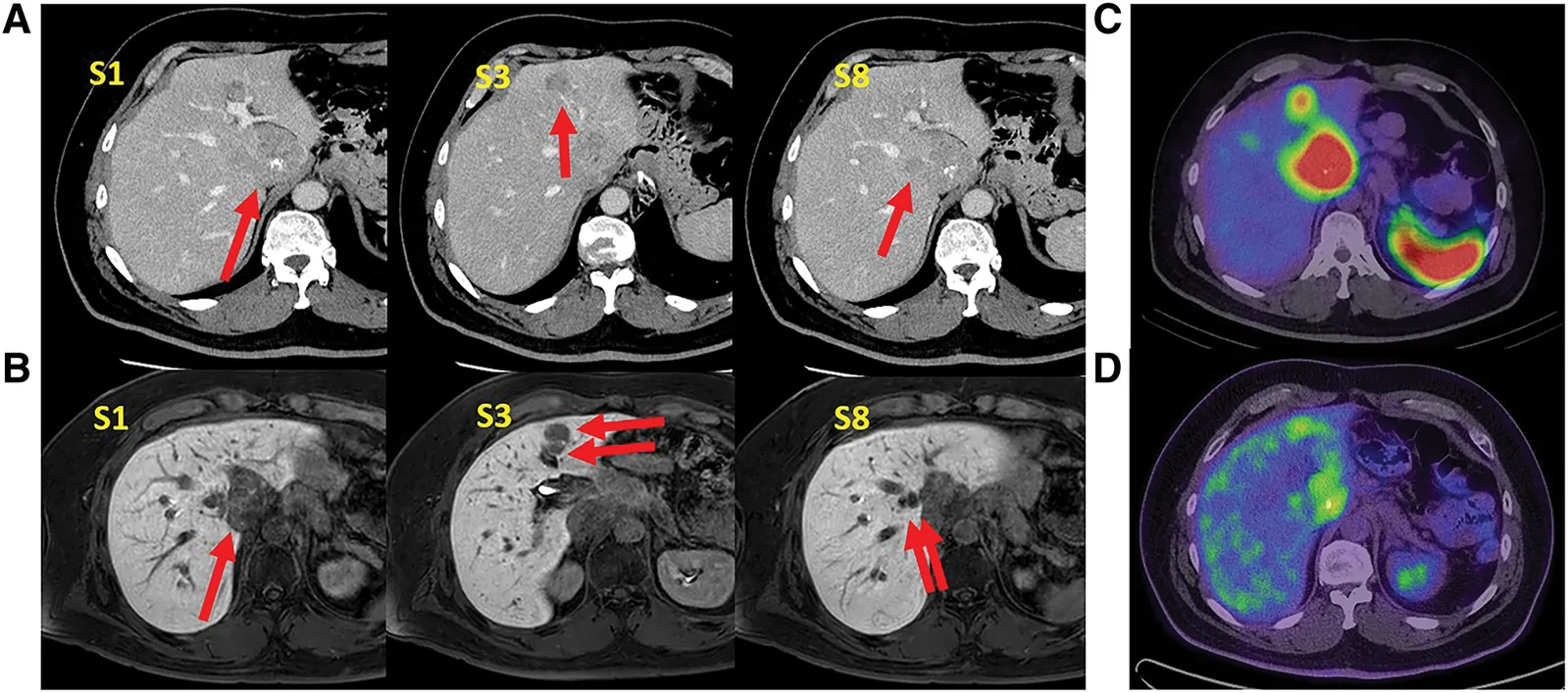
Contrast-enhanced CT and MRI of Case 3. Contrast-enhanced CT revealed 5 early-enhancing nodules measuring 50, 20, 20, 10, and 10 mm: 1 in segment 1, 2 in segment 3, and 2 in segment 8 of the liver (**A**). On Gd-EOB-MRI, these lesions showed decreased uptake in the hepatobiliary phase (**B**). ^111^In-pentetreotide scintigraphy demonstrated abnormal uptake in 1 lesion in segment 1 and 1 lesion in segment 3 (**C**). FDG-PET showed increased uptake in segment 1 (SUVmax, 7.9) and segment 3 (SUVmax, 6.2) (**D**). All arrows indicate tumors. FDG-PET, fluorodeoxyglucose-PET; Gd-EOB-MRI, gadoxetic acid-enhanced MRI; SUVMax, standardized uptake value maximum

**Fig. 6:**
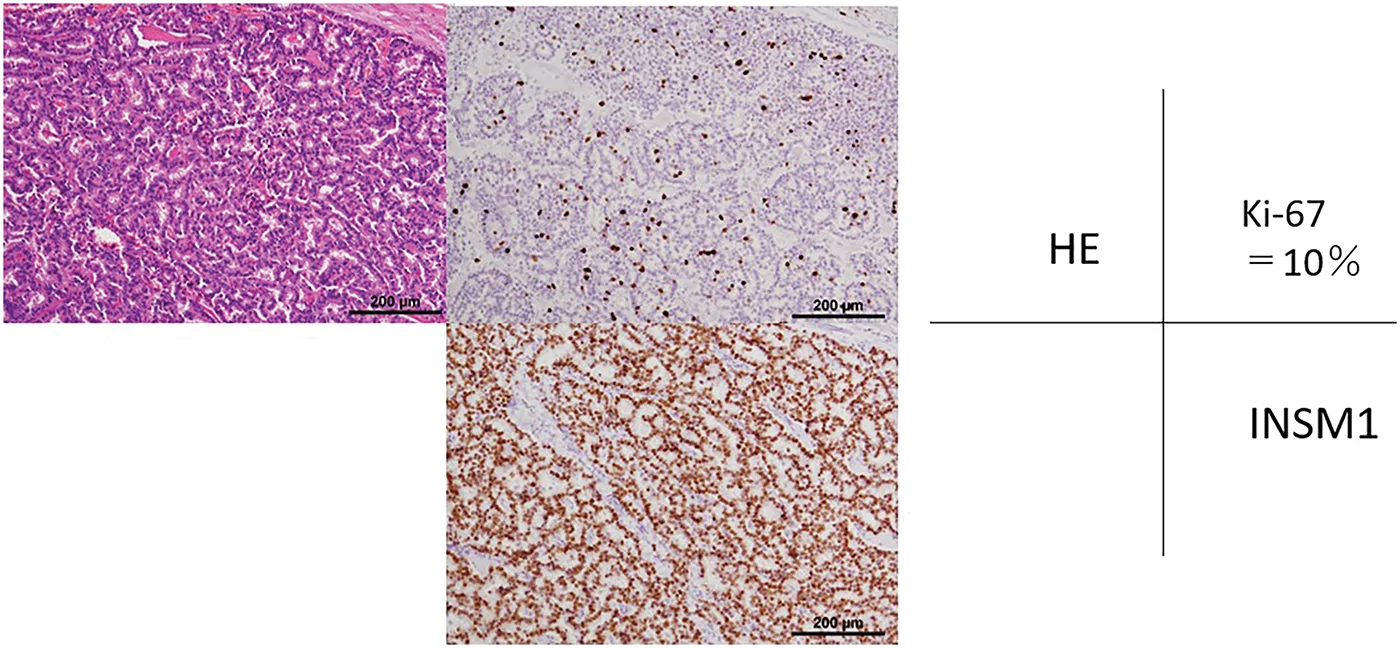
Pathological images of Case 3. Atypical cells proliferated while forming rosette-like and glandular structures. The Ki-67 labeling index was 10%, and immunohistochemistry showed positivity for INSM1. HE, hematoxylin-eosin; INSM1, insulinoma-associated protein 1

## DISCUSSION

NENs are a collective term for tumors predominantly showing neuroendocrine differentiation and most commonly arise in the digestive organs, such as the pancreas and gastrointestinal tract, or in the lungs. Approximately 70% are reported to originate from the pancreas or gastrointestinal tract, 25% from the lungs, and 5% from other organs.^2)^ In Japan, rectal NETs account for more than half of all gastrointestinal NETs.^3)^

In the 2010 WHO classification, all tumors with a Ki-67 index greater than 20% were classified as NEC. In contrast, in the 2022 revised classification, NENs are classified into well-differentiated and poorly differentiated types according to the degree of tumor differentiation, and well-differentiated neoplasms are further classified as NET grade (G) 1–3 based on the Ki-67 index and mitotic count.^4)^ Poorly differentiated NENs are classified as NECs; however, NETs and NECs differ markedly in their histogenesis and molecular behavior and are considered distinct disease entities rather than lesions along the same biological spectrum. Whereas NEC is associated with an extremely poor prognosis, NET may be curable, and long-term survival can be expected with appropriate diagnosis and treatment.^5)^

PHNET is extremely rare, accounting for only 0.3% of all NETs. However, because the liver is a common site of distant metastasis from NENs regardless of the primary organ, PHNET is not usually considered aggressive even when an NEN lesion is identified in the liver. For the diagnosis of PHNET, it is necessary to confirm the absence of a primary lesion in the pancreas or gastrointestinal tract. Nevertheless, even when a tumor is initially considered to be of hepatic origin, an occult minute primary lesion may later become evident, leading to a diagnosis of liver metastasis rather than PHNET.^6)^ For this reason, some authors have suggested that the absence of any detectable primary lesion after at least 3 years of follow-up is necessary to support the diagnosis of PHNET.^7)^ The histogenesis of PHNET remains unclear. Several hypotheses have been proposed, including origin from ectopic pancreatic or adrenal tissue within the liver, neuroendocrine differentiation of the biliary epithelium associated with chronic inflammation, and transformation of hepatic stem/progenitor cells. However, none of these mechanisms has been definitively established.^8)^

In addition, because unresectable neuroendocrine liver metastases have become an indication for deceased-donor liver transplantation in Japan, identification of the primary lesion is also important for determining the appropriate treatment strategy. PHNET remains a diagnosis of exclusion, and occult extrahepatic primary NETs may remain undetected despite extensive evaluation. In the present cases, no extrahepatic primary lesions were identified by upper and lower gastrointestinal endoscopy, contrast-enhanced CT, Gd-EOB-MRI, or functional imaging studies. Pancreatic primary lesions were considered unlikely because no pancreatic abnormalities suggestive of a neuroendocrine tumor were detected on these imaging modalities. However, capsule endoscopy and balloon-assisted enteroscopy were not performed; therefore, the possibility of an occult small bowel primary lesion could not be completely excluded. Ga-68 DOTATATE PET/CT was not available at our institution during the study period, and SRI was performed using ^111^In-pentetreotide scintigraphy. In Case 2, although a gastric submucosal lesion was detected, no abnormal uptake corresponding to this lesion was observed on either ^111^In-pentetreotide scintigraphy or FDG-PET, and no findings suggestive of a gastric neuroendocrine tumor were observed during the clinical course. Therefore, this lesion was considered unlikely to represent the primary site; nevertheless, its origin could not be definitively determined. Accordingly, the diagnosis of PHNET in the present cases should be regarded as tentative even after comprehensive evaluation. Regarding nuclear medicine imaging, SRI is useful because it enables efficient detection of tumors expressing SSTRs. Among SRI modalities, ^111^In-pentetreotide, a SPECT tracer, is currently the only agent covered by insurance in Japan. It should be noted, however, that SRI is not specific for NENs, because positive findings may also be observed in non-NEN tumors with strong SSTR expression, as well as in granulomatous diseases such as sarcoidosis and inflammatory disorders such as rheumatoid arthritis.^9)^ Nevertheless, lesions with a Ki-67 index <15% have been reported to show a high positivity rate of approximately 90% on SRI.^10)^ FDG-PET, which is widely used to detect metastasis and recurrence of malignant tumors, is also useful in NENs, showing a positivity rate of 92% in lesions with a Ki-67 index >15%.^10)^ In other words, well-differentiated NENs tend to be positive on SRI, whereas poorly differentiated NENs tend to be positive on FDG-PET. These modalities are considered to play complementary roles in the detection of gastroenteropancreatic NENs.^11)^ In the present case series, both examinations were performed in all cases. In Case 2, in which the Ki-67 index was approximately 1%, ^111^In-pentetreotide was positive, whereas FDG-PET was negative. In Case 3, in which the Ki-67 index was 10%, both ^111^In-pentetreotide and FDG-PET were positive. In Cases 2 and 3, some lesions showed uptake on ^111^In-pentetreotide scintigraphy and/or FDG-PET, whereas others did not. Because lesion-specific mitotic counts and Ki-67 labeling indices were not evaluated, we could not assess whether the differences in tracer uptake were associated with pathological differences among the lesions. This represents a limitation of the present report. However, interlesional biological heterogeneity may have contributed to the heterogeneous uptake patterns observed on functional imaging.

In the treatment of gastroenteropancreatic NETs, resection of the primary lesion is considered the principle.^12)^ Even in the presence of distant metastasis, surgical resection is recommended when curative resection is feasible, whereas multidisciplinary treatment is recommended for unresectable cases.^13)^ Although clear evidence regarding PHNET is lacking because of the limited number of reported cases, surgical resection has been reported to provide a favorable prognosis.^14)^ In the present case series, surgical resection was performed in accordance with the treatment strategy for gastroenteropancreatic NETs, and all patients are currently alive. However, in Cases 1 and 2, although negative surgical margins were achieved at the initial surgery, recurrence was observed more than 3 years postoperatively, and repeat surgical resection was performed. NENs are known to be prone to recurrence, and the recurrence rate in pancreatic NETs has been reported to be approximately 15%.^15)^ In addition, Hijioka et al.^16)^ reported that tumor size and high grade, defined as NET G2 or higher, were associated with lymph node metastasis and recurrence. According to reports published in Japanese, 23 cases of PHNET treated by surgical resection since 2010, when the classification became more similar to the current system reported, and several reports described postoperative recurrence (**Table 1**).^8,17–37)^ In the present case series, no association was found between tumor size and recurrence. However, all recurrent cases were grade 2 or 3, and no recurrence was observed in grade 1 tumors. In addition, liver metastases from pancreatic NETs are very often diffusely multiple; 1 report showed that 65% of cases had little normal liver parenchyma remaining, and 80% had bilobar disease.^38)^ In contrast, in our review of PHNET, 6 repeat resections were performed for a total of 7 postoperative recurrences in 5 patients. PHNET recurrence appears to be more limited in number and location than liver metastases from NET, suggesting that close follow-up may increase the likelihood of achieving curative resection. In the present series, both the mitotic count and Ki-67 index were higher at recurrence than at the initial presentation in Case 1, whereas in Case 2, the initial tumor had been diagnosed as G2, but the recurrent lesion was classified as G1 because mitotic figures were scarcely observed. It is therefore important to determine the treatment strategy with the possibility in mind that the tumor grade may change at recurrence, and simple observation should be avoided when recurrence is suspected.

**Table 1:** Reported cases of resected PHNET in Japan

No.	Author (year)	Age	Sex	Grade (or Ki-67 index)	Number	Tumor size (cm)	Time to recurrence (month)	Follow-up period (month)	Outcome
1	Yamazaki 2013^17)^	45	F	NETG2 (10%)	1	14	No	12	Alive
2	Yoshida 2013^18)^	38	F	NETG2 (4%)	2	13, 2	No	24	Alive
3	Okumura 2014^19)^	80	M	NETG2 (12.6%)	NR	NR	No	NR	NR
4	Kimura 2014^20)^	57	F	NETG1 (<1%)	1	1.6	No	20	Alive
5	Sakae 2015^21)^	40	F	NETG1 (1.5%)	1	3.6	No	36	Alive
6	Sando 2017^22)^	76	F	NETG2 (5%)	1	8.6	No	8	Alive
7	Sasaki 2018^23)^	55	F	NETG1 (<1%)	Multiple	Max 10	NR	24	Alive
8	Koda 2018^24)^	65	M	NETG2 (11.6%)	1	6.2	61, 76	108	Alive
9	Koda 2018^24)^	72	M	NETG3 (43%)	1	2.5	9	21	Alive
10	Itokawa 2019^25)^	70	F	NETG3 (24%)	1	11	No	15	Alive
11	Hijikawa 2019^26)^	55	M	NETG2 (8%)	NR	NR	108, 132	132	Alive
12	Fujimoto 2019^27)^	70	M	NETG2 (14%)	1	2	No	18	Alive
13	Toda 2019^28)^	53	M	NR	1	7	No	24	Alive
14	Ozawa 2020^29)^	60	F	NETG3 (23.4%)	1	2	No	36	Alive
15	Nishitani 2020^8)^	74	M	NETG1 (1.2%)	1	2	No	30	Alive
16	Tani 2020^30)^	65	F	NETG1 (<2%)	1	2.5	No	6	Alive
17	Yamamoto 2021^31)^	58	F	NETG2 (5%)	1	7	No	10	Alive
18	Onotogi 2022^32)^	40	F	NETG2 (5%)	1	4.1	No	NR	NR
19	Suzuki 2022^33)^	60	M	NETG2 (7%)	3	6.1, 4.8, 1.3	No	NR	NR
20	Murakami 2023^34)^	60	M	NETG2 (8%)	1	5	No	12	Alive
21	Kawahara 2023^35)^	73	M	NETG1 (1.1%)	1	2.7	No	5	Alive
22	Fujii 2024^36)^	44	F	NETG2 (1.2%)	1	2	No	9	Alive
23	Aoyama 2025^37)^	75	F	NETG1 (2%)	1	16	No	12	Alive
24	Case 1	42	F	NETG2 (3.6%)	1	1.8	68	70	Alive
25	Case 2	72	F	NETG2 (1.04%)	3	1.8, 1.5, 0.8	45	52	Alive
26	Case 3	50	M	NETG2 (10%)	5	5, 2, 2, 1, 1	No	6	Alive

F, female; M, male; NETG, Neuroendocrine Tumor grade; NR, not reported; PHNETs, primary hepatic neuroendocrine tumors

In addition, we searched PubMed using the terms “primary hepatic neuroendocrine tumor” and “PHNET” through December 2025. Articles describing surgically treated PHNET with available pathological grading information were included. Conference abstracts, duplicate reports, and cases in which the Ki-67 index or tumor grade was not described were excluded from the analysis (**Table 2**).^39–56)^ As in the cases reported in Japan, the overall prognosis was relatively favorable. Although no recurrence was observed in G1 tumors, recurrence has been reported even 8 years after resection, suggesting that long-term follow-up remains important.

**Table 2:** Reported cases of PHNET treated by surgical resection in PubMed

No.	Author (year)	Age	Sex	Grade (or Ki-67 index)	Number	Tumor size (cm)	Time to recurrence (month)	Follow-up period (month)	Outcome
1	Mitamura 2015^39)^	70	M	NETG1 (2%)	1	2.3	No	24	Alive
2	Hu 2016^40)^	41	F	NETG2 (10%)	1	8.9	No	11	Alive
3	Ibrahim 2017^41)^	57	F	NETG2 (20%)	2	10, 0.7	36	156	Alive
4	Meng 2018^42)^	56	F	NETG1 (<1%)	1	26	No	72	Alive
5	Qu 2018^43)^	72	F	NETG2 (15%)	1	13	No	12	Alive
6	Chen 2019^44)^	65	M	NETG2 (3.4%)	1	7	No	60	Alive
7	Chen 2019^44)^	72	M	NETG1 (0.5%)	1	2	No	6	Alive
8	Jung 2019^45)^	48	M	(0%)	1	12.0	No	264	Alive
9	Jung 2019^45)^	45	F	(0.2%)	1	4.3	No	204	Alive
10	Jung 2019^45)^	37	F	(32.5%)	1	4.0	13	26	Dead
11	Jung 2019^45)^	55	M	(4.8%)	1	4.2	No	171	Alive
12	Jung 2019^45)^	42	F	(1.7%)	1	3.2	No	162	Alive
13	Jung 2019^45)^	64	F	(27.8%)	1	15.0	47	152	Alive
14	Jung 2019^45)^	62	M	(29.0%)	1	8.0	1	5	Dead
15	Jung 2019^45)^	50	M	(45.1%)	1	18.0	2	3	Dead
16	Jung 2019^45)^	48	F	(5%)	1	3.0	23	102	Alive
17	Jung 2019^45)^	26	M	(10%)	1	29.0	5	21	Dead
18	Jung 2019^45)^	48	M	(2%)	1	4.6	No	57	Alive
19	Jung 2019^45)^	70	M	(60%)	1	15.0	1	50	Alive
20	Shah 2019^46)^	35	M	NETG1 (<2%)	1	8.1	No	NR	Alive
21	Shah 2019^46)^	65	M	NETG2 (15%)	2	7.3, 5.2	No	NR	Alive
22	Shah 2019^46)^	62	F	NETG1 (<2%)	1	4.1	No	NR	Alive
23	Zhang 2022^47)^	43	M	NETG2 (5%)	1	5.5	No	NR	Alive
24	Zhao 2022^48)^	26	M	NETG2 (>3%)	1	7.5	No	12	Alive
25	Alghamdi 2023^49)^	60	M	NETG2 (5%)	1	4	No	6	Alive
26	Zhang 2023^50)^	56	F	NETG3 (30%)	Multiple	Max 6.7	No	NR	NR
27	Kushwaha 2023^51)^	73	F	NETG2 (8%–10%)	1	6.9	No	9	Alive
28	Ghattas 2024^52)^	55	M	NETG2 (8%)	2	6.8, 5.3	No	3	Alive
29	Li 2024^53)^	24	F	NETG2 (5%)(recurrent lesion)	1	7.1	96	132	Alive
30	Shidahara 2025^54)^	52	F	NETG2 (4%)	4	NR	No	36	Alive
31	Lv 2025^55)^	67	M	NETG2 (10%)	1	2	No	12	Alive
32	Shao 2025^56)^	70	M	NETG1 (5%)	3	1.5, 1.5, 1	No	6	Alive

F, female; M, male; NETG, Neuroendocrine Tumor grade; NR, not reported; PHNET, primary hepatic neuroendocrine tumor

Adjuvant chemotherapy for patients with risk factors for recurrence has been proposed; however, at present, there is no clear evidence supporting the usefulness of postoperative adjuvant therapy for NENs. Because NENs are rare diseases, the target population, promising regimens, and treatment duration all remain unclear owing to the lack of prospective studies and the presence of conflicting evidence. Postoperative adjuvant therapy is not recommended for gastroenteropancreatic NETs,^57)^ and the same is considered to apply to PHNET.

As mentioned above, liver metastases from NETs are often multiple at the time of diagnosis, making R0 resection difficult, and it has been reported that more than 80% of patients develop intrahepatic or extrahepatic recurrence after surgery.^58)^ Therefore, multidisciplinary treatment, including systemic therapy and PRRT, plays a central role. The ESMO guidelines limit the indication for hepatectomy to tumors with a Ki-67 index of 10% or less or those with slow tumor growth.^59)^ On the other hand, debulking surgery is known to be effective for symptom control in functional tumors, and improvement in overall survival has also been reported even with 70% debulking resection.^60)^ In PHNET and its recurrence as well, when the disease is extensive and R0 resection is difficult, multidisciplinary treatment, including debulking surgery, may be a therapeutic option.

Careful follow-up is essential for the accurate detection of postoperative recurrence. In pancreatic NENs, SRI is recommended every 2 years for G1 tumors and annually for G2/3 tumors, together with CT and MRI.^61)^ Because PHNET is extremely rare, no disease-specific surveillance strategy has been established. However, current guidelines for NETs, including ENETS guidelines, recommend long-term postoperative follow-up using cross-sectional imaging, with the interval determined according to tumor grade, stage, and recurrence risk.^61)^ In addition, previous reports have suggested that PHNET may recur several years after curative resection. In the present series, 2 patients developed intrahepatic recurrence more than 3 years after initial surgery. These findings suggest that long-term surveillance is necessary even after complete resection. Therefore, we performed follow-up with CT and/or MRI every 6 months, particularly considering the rarity of PHNET, the difficulty in predicting its biological behavior, and the potential for late intrahepatic recurrence, and SRI was added at an appropriate time when recurrence was suspected, allowing repeat surgical resection to be performed. In our review of recurrent cases reported in Japan, the median time to recurrence was 61 months, suggesting that follow-up for longer than 5 years is warranted.

In addition, for Cases 2 and 3, mitotic activity was assessed across the available hematoxylin and eosin-stained sections from the multiple hepatic lesions, and the section showing the most prominent mitotic activity was selected for determination of the maximum mitotic count and Ki-67 labeling index. Ki-67 immunohistochemical staining was not performed separately for every individual lesion. Therefore, possible interlesional and intratumoral heterogeneity in the Ki-67 labeling index could not be fully evaluated, which represents a limitation of this study, particularly in interpreting the heterogeneous findings observed on functional imaging.

## CONCLUSIONS

In the present study, we reported 3 resected cases of PHNET, including cases with recurrence several years after the initial surgery, together with a review of the literature. Further accumulation of cases is essential to establish optimal strategies for diagnosis, treatment, and management of recurrent disease.
